# Stem cells in a three-dimensional scaffold environment

**DOI:** 10.1186/2193-1801-3-80

**Published:** 2014-02-11

**Authors:** Xuan Meng, Patrick Leslie, Yanping Zhang, Jiahong Dong

**Affiliations:** Department of Radiation Oncology, University of North Carolina, Chapel Hill, NC 27599-7512 USA; Lineberger Comprehensive Cancer Center, School of Medicine, University of North Carolina, Chapel Hill, NC 27599-7512 USA; Curriculum in Genetics and Molecular Biology, University of North Carolina, Chapel Hill, NC 27599-7512 USA; Department of Pharmacology, School of Medicine, University of North Carolina, Chapel Hill, NC 27599-7512 USA; Hospital & Institute of Hepatobiliary Surgery, Chinese PLA General Hospital, Fuxing Road 28, Haidian District, Beijing, 100853 China

**Keywords:** Stem cells, Cell culture, Tissue engineering, Extracellular matrix, Scaffold

## Abstract

Stem cells have emerged as important players in the generation and maintenance of many tissues. However, the accurate *in vitro* simulation of the native stem cell niche remains difficult due at least in part to the lack of a comprehensive definition of the critical factors of the stem cell niche based on *in vivo* models. Three-dimensional (3D) cell culture systems have allowed the development of useful models for investigating stem cell physiology particularly with respect to their ability to sense and generate mechanical force in response to their surrounding environment. We review the use of 3D culture systems for stem cell culture and discuss the relationship between stem cells and 3D growth matrices including the roles of the extracellular matrix, scaffolds, soluble factors, cell-cell interactions and shear stress effects within this environment. We also discuss the potential for novel methods that mimic the native stem cell niche *in vitro* as well as the current associated challenges.

## Introduction

Stem cells have emerged as important players in the generation and maintenance of many tissues such as fat, nerves, and bones as well as in disease states such as diabetes and cancer (Hay 2013). Embryonic stem cells (ESCs) are pluripotent cells capable of differentiating into all somatic and germ cell types. Although the ESC compartment represents a great resource for cell transplantation and tissue engineering applications, the complexity of environmental factors and other peripheral mechanisms that act on ESC differentiation currently hinder our ability to precisely control them for clinical use. Multipotent mesenchymal stem cells (MSCs), which have restricted differentiation potential, are easier to control than ESCs in terms of differentiation. Another type of stem cell that has shown promise in clinical therapy, bone marrow-derived mesenchymal stem cells (BMSCs) cultured *in vitro* are highly proliferative and can be amplified to 10^9^ cells from a single bone marrow aspirate (personal observations). Furthermore, BMSCs retain a normal karyotype and retain telomerase activity after 12 passages, which is consistent with their observed ability to regenerate with fidelity.

In 2006, Takahashi and Yamanaka reported the successful induction of pluripotent stem cells from somatic cells. These induced pluripotent stem cells closely resemble ESCs in terms of self-renewal and differentiation capacity (Takahashi and Yamanaka 2006), and this research represents a landmark discovery in stem cell research history. Although great success has been achieved regarding the manipulation of stem cells, the production of clinically useful stem cell data requires the appropriate model system. In an attempt to more closely mimic an *in vivo* environment, the culture of ESCs in a three-dimensional (3D) system has been successfully used to learn more about stem cell dynamics such as the assembly of cell adhesions and intercellular signaling during early embryogenesis.

The most important aspects of stem cells are their ability to self-renew and to differentiate into many different kinds of cells. These properties have contributed to the use of stem cells in various ways such as cell replacement therapies (Schulz *et al.*^2012^; Nelander *et al.*^2013^; Sillence *et al.*^2012^), tissue engineering (He and Callanan 2013), and pharmacology or toxicology screens (Desbordes and Studer 2013; Jonsson *et al.*^2012^). Each of these applications requires a large number of cells of high quality, which requires quick cell expansion. Traditional two-dimensional (2D) cultures require stem cell growth to occur in monolayers atop stromal layers that support stem cell proliferation or atop membranes with or without growth factors. Furthermore, 2D culture systems face difficulties in meeting the requirements of many downstream applications due to the inherent heterogeneity, limited scalability or reproducibility (Serra *et al.*^2012^), and incompatibility with the development of *in vitro* models that accurately simulate the native stem cell niche. The precise control of cell behavior is a crucial aspect that must be taken into account when using *in vitro* stem cell models. 3D culture can significantly improve stem cell viability and function offering a higher degree of efficiency, consistency, and predictability to the resulting stem cell manufacturing platform, which also makes the 3D culture system a more promising tool for preclinical research.

Since the 1950s, cells have been cultured on 3D gel substrates. In 1985, Sertoli cell and germ cell survival and differentiation were accomplished by using a 3D substratum*.* Subsequently, many other types of cells (nerve cells, epithelial cells, and endothelial cells) have been successfully cultured using 3D systems (Benton *et al.*^2009^). 3D systems provide useful models for investigating the mechano-biology of how individual cells sense and generate mechanical force in response to their surrounding environment. When fibroblasts interact with 3D collagen matrices, the cells penetrate the matrix and become entangled with matrix fibrils, which promote distinct patterns of signaling and migration. In recent years, Weigelt and Bissell successfully developed a 3D culture system for human mammary cells to investigate various treatments for breast cancer, and subsequently proposed that more intricate 3D models are required to fill the gap between 2D cell culture systems and whole-animal systems (Weigelt and Bissell 2008). Ample evidence has been published suggesting the importance of 3D growth system for stem cells. For example, chondrocytes tend to lose their rounded morphology in 2D culture and assume a fibroblast-like morphology, which is accompanied with changes in the biosynthesis of matrix proteins; however, after introducing the differentiated chondrocytes grown in a 2D system into a 3D system, these cells can re-differentiate into rounded chondrocytes (Lawrence and Madihally 2008). Rhee 2009have proposed an explanation for the differences between 2D and 3D systems by suggesting that signaling pathways for matrix remodeling in fibroblast-3D collagen matrices showed that differences in cell signaling, cytoskeleton modulation, and integrin-focal dynamics during promigratory processes are critical for cell mechanics and cell-matrix interactions Rhee (2009). 3D culture systems have also been successfully used to generate tissue as reported by Koehler *et al.*^2013^, who successfully generated inner ear sensory epithelium from pluripotent stem cells by using a 3D culture system (Koehler *et al.*^2013^). In this review, we discuss the advantages of stem cell culture using 3D systems with an emphasis on recent examples in the field that demonstrate a field-wide transition from 2D culture to 3D culture.

### Three-dimensional stem cell culture

There are many types of 3D stem cell culture systems, which can include plate or culture dish, spinner flask, rotating wall vessel (RWV), and perfusion bioreactor system among others. The plate or culture dish is the most widely used system because it is low-cost and easy to use. However, some major disadvantages of this system include low seeding efficiency, an inherent limit on growth inside of the dish and a vastly different environment when compared with an *in vivo* setting. When compared with the culture dish system, the spinner flask and rotating wall vessel offer improvements with respect to the quality and efficiency of cell culture, as they promote the convection of the culture medium by stirring. The RWV is composed of two horizontal concentric cylinders, and the gas inside the column can be freely exchanged through a semi-permeable membrane. The perfusion bioreactor system has become more popular in stem cell engineering projects. The perfusion bioreactor system maintains a balanced environment by constantly refreshing the culture solution thus reducing the likelihood of contamination. Despite the advances in stem cell 3D culture systems, investigators continue to identify new methods to culture cells more efficiently.

Stem cells can also be cultured under several different conditions including as cell aggregates (Singh *et al.*^2010^; Zweigerdt *et al.*^2011^; Amit *et al.*^2011^), in the presence of microcarriers (Chen *et al.*^2011^; Storm *et al.*^2010^), on alginate microencapsulates (Serra *et al.*^2011^; Jing *et al.*^2010^), in thermoreversible hydrogel (Lei and Schaffer 2013), and in nanostructure scaffolds composed of self-assembling peptides (Cunha *et al*. 2013; Gelain *et al*. 2006) among others*.* These culture conditions share the advantages of ease of use, scalability, and reproducibility, although each of these growth methods also has distinct advantages. Cell aggregate growth systems cost less than the others, as cell aggregate systems do not require additional materials. Microcarrier systems can produce cells of better quality and purity because this system possesses good mass and gas diffusion properties. Alginate microencapsulates and thermoreversible hydrogel systems offer protection to cells from shear force-induced cell death. Cells grown in thermoreversible hydrogel present with the highest expansion rate of these growth methods, as thermoreversible hydrogel has been shown to expanse to 6.4 × 10^7^ folds after 30 days (Lei and Schaffer 2013). Nanostructure scaffolds composed of self-assembling peptides have the ability to form a biologically active matrix that displays functional motifs such as RGD (arginine-glycine-aspartic acid), BMHP1 (bone marrow homing peptide 1), and BMHP2 (bone marrow homing peptide 2).

### Stem cell differentiation in 3d systems

Although significant advances have been made recently in the development of artificial kidneys, pancreata, livers, cardiac muscle, skeletal muscle, and blood vessels (Gong and Niklason 2008), a better understanding of the cellular mechanisms that guide stem cell behavior in native and engineered 3D microenvironments would facilitate even greater progress. Current efforts aim to provide proper 3D structural, biochemical, mechanical, and stimulatory environments for stem cells.

Mature cells cultured in 3D matrices exhibit altered phenotypes that inhibit their proliferative nature and enhance their ability to form higher order structures. Furthermore, mature cell growth in a 3D matrix enhances stem cell potential by providing the dynamic interface that naturally occurs between the stem cells and the matrix. For example, in an angiogenesis study by Benelli and Albini, the authors found that vascular endothelial cells could form capillary-like structures with a lumen when cultured on basement membrane gels. Benton *et al.*^2009^further showed that cell density and time affect the morphology/differentiation of vascular endothelial cells grown on 3D basement membranes (Benton *et al.*^2009^). Additional evidence has suggested that differentiated cells that were cultured in 3D matrices can be more readily transplanted into animals for further investigation. For example, pluripotent stem cell-derived hepatocyte transplantation led to a significant increase in the survival rate and a reduction in liver damage in mice model (Vosough *et al.*^2013^; Khanjani *et al.*^2013^). In another study, Mukai *et al.*^2008^reported the use of a 3D cell culture system to identify endothelial progenitor cells (Mukai *et al.*^2008^), which offered a significant technological advance for investigating cell-based therapies for injured and ischemic tissues.

3D *in vitro* differentiation studies have a significant advantage in that they can be used to more accurately determine the function of any gene through mutation or knockout (Komura *et al.*^2008^; Wu *et al.*^2007^). ESCs first aggregate into embryoid bodies (EBs), and then a layer of primitive endoderm (PE) forms on the exterior surface of the EBs, which is dependent on fibroblast growth factor (FGF) signaling mediated by the PI3-kinase pathway. The PE cells develop further into the endoderm and deposit a basement membrane rich in laminin and collagen IV (Bratt-Leal *et al.*^2009^). The development of EBs has been explained in detail by Bratt-Leal *et al.*^2009^*,* and the entire process appears to be influenced by EB formation and culture methods. Although much about the EB formation process is known, computational modeling used to predict stem cell differentiation behavior in a 3D system suggests that stem cell fate specification within EBs is a poorly controlled process (White *et al.*^2013^).

Factors within the extracellular environment have been shown to influence the differentiation of stem cells, which has led investigators to focus on the extracellular matrix (ECM), growth scaffolds, soluble growth factors, shear stress effects, and other components of the extracellular environment.

### Extracellular Matrix (ECM)

The ECM is a structural skeleton that provides mechanical support and influences the development of various stem cell phenotypes. The complex architecture of proteins, polysaccharides, and proteoglycans provides stem cells with a microenvironment that influences their pattern of growth and development. Based on the fact that ESCs form EBs, soluble molecules such as collagen and laminin must be added to suspensions of ESCs during EB formation to manipulate the inner microenvironment of the EBs (Bratt-Leal *et al.*^2009^).

Unfortunately, in most cases, analysis of the effects of widely variable and complex growth and differentiation signals within synthetic ECMs is not practical. Therefore, novel engineering approaches are needed to effectively examine the effects of specific ECM-derived signals and signal combinations on stem cell behavior. As a result, ‘enhanced’ spatially patterned ECMs have been developed. Goh *et al*. 2013have compared the biological nature of the ECM with respect to EBs after different treatments and have shown that decellularized ECMs from spontaneously differentiated EBs provide a more favorable microenvironment that promotes ESC attachment, proliferation, and early differentiation relative to native EBs (Goh *et al.*^2013^). This study suggests that ESC differentiation depends on the appropriate ECM scaffold, which could have major implications for tissue engineering applications. In another study investigating ECM properties, Lund *et al.*^2009^demonstrated a role for discoidin domain receptor 1 (DDR1) in the stem cell response to and interaction with type I collagen within a 3D growth system. Inhibition of DDR1 decreased osteogenic potential while increasing cell spreading, stress fiber formation, and ERK1/2 phosphorylation. Furthermore, inhibition of DD1 was shown to alter the cell-mediated organization of a naive type I collagen matrix, which represents an important component of the bone matrix (Lund *et al* . 2009). Jongpaiboonkit *et al.*^2008^*.* Developed an automated approach to generate polyethylene glycol (PEG) hydrogel arrays, which were designed to present a widely adaptable range of ECM-derived signals to multiple cell types in a 3D context (Jongpaiboonkit *et al.*^2008^). Subsequently, Jongpaiboonkit *et al.*^2008^used their 3D PEG hydrogel arrays as a platform to screen for the individual and combinatorial effects of multiple ECM parameters on human MSC (hMSC) viability, cell density, ECM degradation, cell adhesion ligand type, and cell adhesion ligand concentration. Their results suggest that hydrogel degradation increases hMSC viability due to the enhanced mass transport in the degraded hydrogel networks and decreased physical confinement of the hMSCs.

### Scaffolds

The use of 3D scaffolds in tissue engineering is the most common stem cell culture method. Stem cells can form cell/scaffold structures through a variety of methods upon seeding in 3D scaffolds, and under the proper conditions, the cells undergo proliferation, differentiation, and secretion of specific ECM molecules, which can form additional scaffold and promote cell adhesion and proliferation *in vivo. In vitro* scaffolds can play a role as an *in vivo* matrix by guiding angiogenesis pathways to vascularize newly formed tissue. The ideal scaffold should be 3D, porous with an open pore network, biocompatible, biodegradable, and mechanically amenable to proper cell growth.

In a review by Lawrence and Madihally 2008, the authors summarize the main factors that affect cell colonization of 3D porous scaffolds including 3D architectural features of the porous structure (porosity, pore size, fiber orientation, pore interconnectivity, and topography), scaffold stiffness, and cell-structure interactions (cellular adhesion, mechano-transduction, and matrix turnover) (Lawrence and Madihally 2008).

There are 5 major types of scaffold materials that are currently used: (1) metals (such as titanium, although few metallic scaffolds are used due to the lack of degradability) (Ryan *et al.*^2009^; Elliott *et al.*^2012^), (2) synthetic organic materials (polymers and copolymers) (Tseng *et al.*^2013^; Lakshmanan *et al.*^2013^), (3) synthetic inorganic materials (hydroxyapatite) (Wei *et al.*^2013^; Schumacher *et al.*^2013^), (4) natural organic materials (collagen, fibrin, and hyaluronic acid) (Campbell *et al.*^2011^; Shoae-Hassani *et al.*^2013^), and (5) natural inorganic material (coralline hydroxyapatite) (Rosa *et al.*^2008^; Mygind *et al.*^2007^). Hanjaya-Putra and Gerecht have provided a detailed review of the different characteristics of each kind of scaffold. One scaffold material that is suitable for *in vivo* transplantation is soft 2% methylarcylate HA hydrogel, which is associated with the disadvantage of degradation upon *in vitro* cell culture and thus presents a challenge for long term *in vitro* cultures. Alginate scaffolds are effective for *in vitro* EB formation because of their large pore size and good mechanical properties. In conclusion, the use of scaffolds seeded with stem cells has the potential to serve a large range of tissue engineering applications.

### Soluble factors

Soluble factors play an important role in directing stem cell fate (Taylor-Weiner *et al*. 2013; Lu *et al*. 2013; Hannum *et al*. 1994). Soluble factors bind to cell surface receptors and activate downstream pathways that influence stem cell development and differentiation. 3D-cultured cells differ in cell shape relative to cells cultured in 2D, which can in turn influence the interactions between the ECM and the cell membrane. For example, when induced to differentiate in restrictive ECM environments, adhesive, flattened hMSCs preferentially adopt an osteogenic phenotype, whereas round hMSCs preferentially undergo adipogenesis (McBeath *et al.*^2004^).

Soluble factors retained in the matrix also significantly influence cell fate to varying degrees depending on the cell type and the complement of genes expressed by the cell. Soluble factors have been a point of focus for several studies using 2D model systems, which has contributed to our understanding of cell-matrix interactions in 3D environments. Small molecules such as ascorbic acid, retinoic acid, and dexamethasone as well as larger molecules such as fibroblast growth factors, bone morphogenic proteins, and transforming growth factors comprise examples of soluble factors that affect ESC differentiation. Another consideration is that each cell cultured in a 3D system is not exposed to the same concentration of soluble factors. For example, the formation of EBs permits only the exterior cells to come into direct contact with soluble factors present within the culture medium. Although the studies discussed above demonstrate that stem cell behavior can be regulated by controlled exposure to signaling molecules, delivery of the optimal signaling components required to affect stem cell viability, lineage-specific differentiation, and tissue formation to the desired extent remains a considerable obstacle.

### Cell-cell interactions

Many types of cell-cell interactions have been implicated in a variety of cell fate decision processes during development and adult tissue morphogenesis. Previous studies have shown that Notch signaling ligand (Jagged1) (Beckstead *et al.*^2006^) and the cell adhesion molecule N-cadherin effectively modulate the balance between cell-cell and cell-matrix interactions resulting in significant changes in the overall spatial remodeling that occurs during stem cell differentiation. In a review by Bratt-Leal *et al.*^2009^, the authors explain in detail several of the mechanisms involved in cell–cell interactions, which are mediated primarily by cadherins, in terms of their control over EB differentiation (Bratt-Leal *et al.*^2009^). In a study performed by Parekkadan *et al.*^2008^, the authors explored the role of cell-cell interactions during neuroectodermal specification of ES cells by using a microfabricated cell pair array. The results of this study showed that the expression of connexin (Cx)-43 correlated with the neuroectodermal specification and lineage commitment, suggesting that cell-cell interactions are indeed critical for the control of stem cell differentiation (Parekkadan *et al.*^2008^). Additional studies have provided further evidence for the importance of cell-cell interactions with respect to stem cell differentiation in a wide variety of cell types. One study has shown that enhancement of osteogenesis from MSCs requires ephrinB2, which is a cell surface anchored ligand that specifically interacts with cells expressing the cognate EphB4 receptor through direct contact (Tierney *et al.*^2013^). In another study by Wagner *et al.*^2007^, the authors investigated the adhesive interaction of various fractions of hematopoietic progenitor cells (HPC) by separating them into adherent and nonadherent cells. They found that self-renewing capacity was significantly higher in the adherent fraction than in the nonadherent fraction, and that genes coding for adhesion proteins and extracellular matrix proteins were more highly expressed in the adherent fraction (Wagner *et al.*^2007^).

### Shear stress effects

Cells are affected by a range of mechanical forces during embryogenesis, and those external forces such as flow shear stress are important for the transformation and differentiation of the embryo (Mammoto and Ingber 2010; Krieg *et al.*^2008^). Flow shear stress can be defined as the frictional force generated by the movement of fluid on a surface. In other words, flow shear stress refers to the stress that a moving fluid applies tangential to the solid boundary of an object. Shear stress is measured as the force per unit area typically expressed in units of dynes/cm^2^. Flow shear stress plays a prominent role in stem cell differentiation. In one study performed by Huang *et al.*^2005^, mouse ESCs were cultured on the lumenal surface of a microporous tube with a compliance similar to that of a human artery. When cultured in the presence of differentiation medium that remained static, the ESCs largely presented with a phenotype consistent with smooth muscle cells. However, when the ESCs were cultured in differentiation medium that was flowed through the tube in a weak pulsatile manner simulating venous blood flow, the ESCs exposed to the lumen differentiated into endothelial-like cells, whereas ESCs found within the interstices of the tube walls differentiated into smooth muscle-like cells (Huang *et al.*^2005^). In another similar study, Dong *et al.*^2009^showed that shear stress significantly increased the expression of endothelial cell marker levels including platelet-endothelial cell adhesion molecule-1 (PECAM-1), VE-cadherin, and CD34 at the mRNA and protein levels in MSCs when compared with non-stressed cells (Dong *et al.*^2009^). Different shear stress levels could also influence stem cell differentiation in different ways. MSC migration ability appears to be induced through mitogen-activated protein kinase (MAPK) pathways under lower shear stress (0.2 Pa), whereas MSC migration ability under high shear stress (>2 Pa) is largely inhibited (Yuan *et al.*^2013^). Stolberg and McCloskey have recently reviewed studies that support the role of shear stress during different stages of endothelial differentiation and osteogenesis, and have suggested that stem cells could sense flow shear stress through integrin-mediated signaling, membrane fluidity, ion channels, G-protein-coupled receptors, endothelial glycoalyx, and primary cilia. The primary cell responses include dimerization, recruitment, and colocalization of surface receptors, activation of a variety of tyrosine kinases and intracellular signaling cascades, and nuclear translocation leading to the upregulation or downregulation of a variety of gene products resulting in osteoskeleton reorganization (Stolberg and McCloskey 2009). Collectively, these studies support the potential of using flow shear stress as a differentiation tool, which, in conjunction with other extrinsic factors (e.g., growth factors and extracellular matrix), can effectively modulate the biology of cultured stem cells (Adamo and Garcia-Cardena 2011).

### Prospection of 3d culture systems

Although 2D stem cell differentiation systems have been successfully used to spatially and temporally control the expression of molecules involved in cell differentiation, differentiation also requires the synergistic effects of cell-cell and cell-ECM interactions that can only be provided in the context of 3D systems.

The use of microRNAs (miRNAs), which refers to a class of small, non-coding RNAs that can suppress the expression of mRNAs by binding to the 3′ untranslated region of specific transcripts, to direct stem cell differentiation and function in 3D systems has become a popular method used to study stem cell differentiation. Kim *et al.*^2006^showed that miRNA-206 transfected into myoblasts (muscle cell precursors) can stimulate cell viability, growth, and proliferation without the presence of serum components, and that inhibition of the miRNA-206 with an antisense oligonucleotide inhibits cell cycle withdrawal and differentiation (Kim *et al.*^2006^). In ESCs, the transcriptional and epigenetic networks are also controlled by miRNAs (Melton and Blelloch 2010). For example, miRNA-145 represses Oct4, Sox2, and Klf4 expression (Xu *et al.*^2009^). Likewise, miRNAs-134, 296, and 470 have been shown to target the coding sequence of mouse Nanog, Oct4, and Sox2 (Tay *et al.*^2008^), and miRNA let-7e has been shown to modulate early nephrogenic marker expression and the Wnt pathway, which is an important stem cell signaling pathway (Vinas *et al.*^2013^). In the presence of miRNA-processing enzyme deficiency such as in Dgcr8 knockout mice, differentiation defects are observed in ESCs (Wang *et al.*^2007^). Furthermore, upon transfection into MSCs, certain miRNAs can regulate neurotransmitters to direct neuronal cell differentiation in 3D hydrogel growth environments (Salinas and Anseth 2009; Greco and Rameshwar 2007). In human MSC cultures, miRNA-148b mimics sensitized hMSCs to soluble osteogenic factors resulting in a rapid and robust induction of bone-related markers including alkaline phosphatase activity and calcium deposition. More importantly, miRNA transfection increases osteogenic markers in 3D tissue scaffolds, suggesting that controlling miRNA activity in MSCs can be an effective tool for enhancing the induction of osteogenesis for tissue engineering purposes (Mariner *et al.*^2012^). Collectively, these studies show promise in the area of directing stem cell function and fate in a 3D environment using miRNA technology. Thus, the use of miRNAs particularly in 3D cell culture systems will continue to increase our knowledge of factors involved in stem cell differentiation and development.

Developing novel methods to test mechanistic hypotheses in engineered 3D microenvironments will improve our ability to reconstitute the native stem cell niche in a manner that allows the development of functional tissue products in a more predictable, consistent, and safe manner. Furthermore, *in vitro* remodeling using complex 3D matrix culture systems will provide important advances that will help resolve the current challenges in stem cell growth and differentiation and tissue engineering.

### Ethics statement

This and related studies all complied with the Declaration of Helsinki. Informed consent was obtained from all subjects before they were enrolled in our study. The related animal study proposal was approved by the Institutional Animal Care and Use Committee (IACUC) of the University of North Carolina, USA with the permit number 13-044.
